# Early Prediction of Necrotizing Pneumonia in Children with Mycoplasma Pneumoniae Pneumonia: Development and Temporal Validation of a Clinical Model

**DOI:** 10.3390/children13040473

**Published:** 2026-03-29

**Authors:** Ying Lu, Yushun Wan, Na Zang

**Affiliations:** 1 Department of Respiratory, Children’s Hospital of Chongqing Medical University, National Clinical Research Center for Children and Adolescents’ Health and Diseases, Ministry of Education Key Laboratory of Child Development and Disorders, Chongqing Key Laboratory of Child Rare Diseases in Infection and Immunity, Chongqing 400014, China; yinglu00012@163.com; 2 College of Basic Medicine, Chongqing Medical University, Chongqing 400016, China; yswan@cqmu.edu.cn

**Keywords:** *Mycoplasma pneumoniae*, necrotizing pneumonia, clinical prediction model, machine learning, pediatrics

## Abstract

**Highlights:**

**What are the main findings?**

- We developed and temporally validated a five-predictor model for necrotizing pneumonia in children hospitalized with Mycoplasma pneumoniae pneumonia using routinely available early clinical data.
- The model showed good discriminatory performance in both the development cohort and the later validation cohort, supporting its potential use for early risk stratification.

**What are the implications of the main findings?**

- This model may help identify children at higher risk before overt necrotizing changes become evident on imaging, which may support closer monitoring and earlier reassessment.
- Because it relies on routinely available variables, the model may be more feasible for future clinical translation, although further external validation is still required.

**Abstract:**

Background: Necrotizing pneumonia is a severe complication of *Mycoplasma pneumoniae* pneumonia (MPP) in children. Early recognition remains challenging because initial clinical manifestations are often non-specific, highlighting the need for a practical tool for early risk stratification. Methods: We conducted a single-center retrospective study of hospitalized children with MPP. Data from 2015–2023 were used for model development, and patients enrolled in 2024 were reserved for temporal validation. We compared candidate machine-learning algorithms and selected a parsimonious random forest model using routinely available variables obtained during the early hospitalization period. Model performance was evaluated using discrimination, calibration, and decision curve analysis, and model interpretability was assessed using SHapley Additive exPlanations (SHAP). Results: The random forest model showed good discriminatory performance in internal validation and retained acceptable performance in the 2024 temporal cohort. Calibration indicated reasonable agreement between predicted and observed risks. Decision curve analysis suggested potential clinical value as a supportive tool for early risk stratification. SHAP analysis highlighted fever duration, C-reactive protein, pleural effusion, alanine aminotransferase, and gamma-glutamyl transferase as the main contributors to model prediction. Conclusions: We developed and temporally validated a clinical prediction model for necrotizing pneumonia in children hospitalized with MPP. The model may support early risk stratification using routinely available clinical data, but it is intended to complement rather than replace clinical judgment. External prospective validation is required before routine clinical implementation.

## 1. Introduction

*Mycoplasma pneumoniae* is one of the major causes of community-acquired pneumonia in children and adolescents. Its circulation often shows cyclical epidemic activity, with outbreaks recurring every few years [1,2,3,4]. At the same time, macrolide resistance has become increasingly common in many regions [5,6]. In some areas, reported resistance rates exceed 90%, which further limits treatment options [7]. During the COVID-19 pandemic, non-pharmaceutical control measures reduced the transmission of many respiratory pathogens, including *Mycoplasma pneumoniae*. After these restrictions were relaxed, case numbers rose again in China in 2023. This rebound was not simply a return to pre-pandemic levels. Higher epidemic peaks were observed, severe presentations became more frequent, and younger children appeared to be affected more often than before. Several factors may have contributed to this pattern, including reduced population immunity after the pandemic, possible changes in circulating strains, and the continuing selective pressure from antibiotic use [8,9,10,11,12,13].

Although most children with Mycoplasma pneumoniae pneumonia recover without major sequelae, a subset develop MPP-associated necrotizing pneumonia (MPNP), which represents a far more destructive form of lung injury. This condition is characterized by parenchymal necrosis and the formation of multiple thin-walled cavities within areas of consolidation [14]. Imaging often shows well-defined cavitary lesions with little or no peripheral enhancement, which helps distinguish MPNP from other pulmonary conditions. In clinical practice, affected children may present with persistent high fever and worsening productive cough. Respiratory distress may worsen as the disease progresses. In some cases, the clinical course deteriorates rapidly and leads to serious complications, including hydropneumothorax [15,16,17]. Recent studies have suggested that MPNP is becoming more common, particularly among children with refractory MPP [18,19,20,21]. A large multicenter study from China further showed that Mycoplasma pneumoniae was among the most frequent pathogens associated with necrotizing pneumonia in children, with a higher prevalence reported in northern regions than in southern areas [22].

When the condition progresses to necrotizing pneumonia, lung damage can progress rapidly. Extensive liquefactive necrosis can occur, followed by cavitary destruction of the affected lung parenchyma. In more severe cases, acute respiratory distress syndrome and sepsis may also arise. Compared with children who have uncomplicated MPP or typical pneumonia, those with MPNP are more likely to require oxygen support and intensive care, and their pulmonary recovery is often prolonged. The burden of MPNP therefore extends beyond the acute stage and may affect later respiratory function as well as overall quality of life [23].

Early recognition of MPNP remains difficult because the initial manifestations are often subtle and non-specific. In many children, the more typical imaging findings do not become evident until the disease has already advanced. This gap between early clinical presentation and later radiologic confirmation creates a clear need for tools that support earlier risk assessment. In this setting, the main value of a prediction model is not to replace clinical judgment or to predict the effect of a specific intervention. Its role is to help identify children who may need closer monitoring, earlier reassessment, and more timely imaging or supportive evaluation during hospitalization.

Against this background, we aimed to develop and temporally validate a clinical prediction model for necrotizing pneumonia in children hospitalized with MPP using routinely available early clinical variables. We also sought to compare candidate machine-learning algorithms and to improve interpretability through SHAP, so that the model could be understood more easily in a clinical context.

## 2. Materials and Methods

### 2.1. Study Design and Population

We conducted a retrospective study of children hospitalized with MPP at the Children’s Hospital of Chongqing Medical University between January 2015 and December 2024. During this period, 19,710 children met the diagnostic criteria for MPP. Among them, 156 developed necrotizing pneumonia, corresponding to an overall prevalence of 0.79% in the source population. Hospitalizations from 2015 to 2023 were used for model development, whereas those from 2024 were reserved exclusively for temporal validation and were not involved in predictor selection or model training.

MPP was diagnosed in children who had clinical findings consistent with pneumonia together with microbiological evidence of Mycoplasma pneumoniae infection. Etiologic confirmation required at least one of the following: a positive Mycoplasma pneumoniae immunoglobulin M (MP-IgM) result by particle agglutination at a titer of ≥1:160; a fourfold or greater change in Mycoplasma pneumoniae immunoglobulin G (MP-IgG) titer between paired serum samples collected 2–3 weeks apart; detection of MP DNA or RNA in respiratory samples or bronchoalveolar lavage fluid by real-time PCR; or isolation of Mycoplasma pneumoniae by conventional culture.

Children were excluded if they had major underlying conditions that could affect immune function or alter lung structure. These conditions included congenital heart disease, malignancy, immunodeficiency, connective tissue disease, and hematologic disorders. We also excluded children admitted during the convalescent stage of pneumonia. In addition, patients with congenital or chronic pulmonary diseases, including bronchopulmonary dysplasia, airway malformations, or active pulmonary tuberculosis, were not included [24].

### 2.2. Definition of Necrotizing Pneumonia

Necrotizing pneumonia was defined by characteristic imaging changes identified during hospitalization. Initial chest radiography or computed tomography usually showed extensive pulmonary consolidation. Follow-up imaging then demonstrated liquefactive necrosis within the consolidated parenchyma. Typical findings included single or multiple thin-walled, or even wall-free, cavities within the affected lung. Vesicle-like lucencies or focal areas of low attenuation were also considered supportive features. The presence of an air–fluid level provided additional support for the diagnosis of necrotizing pneumonia [25].

### 2.3. Propensity Score Matching

Given the low frequency of necrotizing pneumonia in the source population, we constructed matched case–control datasets within each study period. In the development period (2015–2023), 76 children with necrotizing pneumonia were matched with 151 children without necrotizing pneumonia. In the temporal validation period (2024), 80 necrotizing cases were matched with 160 non-necrotizing controls. Matching was performed separately in the two periods so that the validation dataset remained independent of the development process.

Propensity scores were estimated using age (months) and body weight, while sex was controlled through exact matching. Nearest-neighbor matching was then performed with an intended 1:2 ratio and a caliper width of 0.05. Owing to the caliper restriction, a small number of cases could not be matched to two eligible controls, resulting in minor deviation from the target ratio in the final matched dataset. All matching procedures were performed in R (version 4.4.3; R Foundation for Statistical Computing, Vienna, Austria).

**Figure 1:**
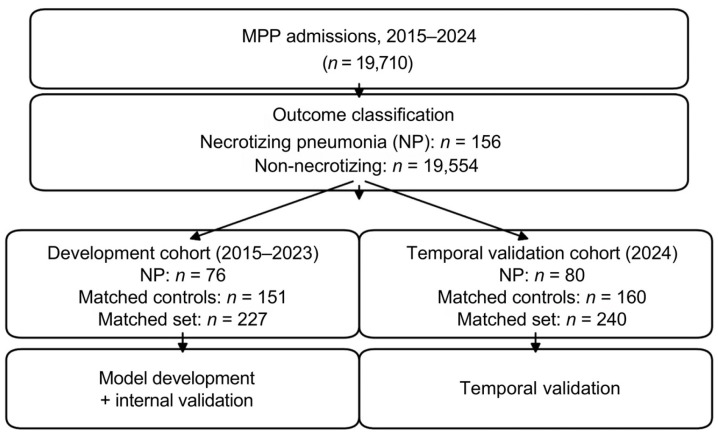
The overall study workflow. MPP, Mycoplasma pneumoniae pneumonia; NP, necrotizing pneumonia.

The matched dataset from 2015 to 2023 was used for model development, whereas the matched 2024 dataset was reserved exclusively for temporal validation. This approach was chosen to improve case–control comparability in the context of a rare outcome, while maintaining a temporally independent validation set. The overall study workflow is presented in Figure 1.

### 2.4. Clinical Data Collection

Clinical, laboratory, and imaging data were extracted from the electronic medical record system. Candidate predictors were selected from variables that were routinely available during the early hospitalization period and were intended to reflect information that would typically be available before necrotizing changes became evident on imaging.

Missing data were limited across all candidate predictors in both cohorts, with each variable showing less than 5% missingness (Supplementary Table S1). Because the overall proportion of missing data was low, the analyses were performed using complete cases.

Because this was a retrospective study based on routinely recorded electronic medical records, blinding was not applicable to predictor assessment or outcome classification.

### 2.5. Predictor Coding and Handling

All candidate predictors were entered into the feature-selection and model-development procedures using their original recorded forms whenever possible. The initial candidate set included routinely collected clinical, imaging, and laboratory variables available during the early hospitalization period (Supplementary Table S2), including commonly measured laboratory indicators such as procalcitonin (PCT), lactate dehydrogenase (LDH), fibrinogen, white blood cell count, neutrophil percentage, lymphocyte percentage, C-reactive protein (CRP), D-dimer, prothrombin time (PT), thrombin time (TT), alanine aminotransferase (ALT), and gamma-glutamyl transferase (GGT). Continuous laboratory variables were analyzed as continuous measurements rather than being dichotomized as normal or abnormal whenever possible. Fever duration was recorded in days. Pleural effusion was treated as a binary imaging variable based on its presence or absence during the early hospitalization period. No longitudinal updating of predictors was performed; therefore, the model was based on baseline or early in-hospital data rather than serial dynamic measurements.

### 2.6. Feature Selection

Statistical analyses, feature selection, and model development were performed using Python (version 3.12.9; Python Software Foundation, Wilmington, DE, USA). Feature selection was performed using only the development cohort from 2015 to 2023. Although a broader set of candidate variables was examined during the initial analysis (Supplementary Table S2), only predictors that were retained consistently across the predefined feature-selection procedures were entered into the final models. To reduce reliance on any single selection strategy, we applied four complementary approaches: L1-regularized logistic regression, random forest feature-importance ranking, recursive feature elimination based on logistic regression, and support vector machine–based recursive feature elimination.

Variables retained by at least three of the four methods were considered relatively stable and were included in an extended candidate model. This process identified eight variables: ALT, CRP, GGT, D-dimer, PT, TT, fever duration, and pleural effusion.

Variables selected consistently by all four methods were considered the most robust candidates and were used to build a more parsimonious primary model. This primary model included ALT, CRP, GGT, fever duration, and pleural effusion.

### 2.7. Model Development

Model development was performed using the matched development cohort from 2015 to 2023. We compared six candidate algorithms: logistic regression, random forest, extreme gradient boosting, support vector machine, k-nearest neighbors, and decision tree. Hyperparameters were tuned by Bayesian optimization within five-fold stratified cross-validation, using the area under the receiver operating characteristic curve (AUC) as the optimization metric.

To avoid depending on a single random split of the data, predicted probabilities for all patients in the development cohort were generated by out-of-fold (OOF) cross-validation. These OOF predictions were then used to determine the classification threshold. The threshold was selected by maximizing the F1 score, which balances precision and recall.

After hyperparameter tuning and threshold selection, each model was refitted using the full development cohort. The final models, together with their prespecified thresholds, were then evaluated in the temporally independent 2024 validation cohort.

The primary analysis was based on the parsimonious five-feature set, which included alanine aminotransferase, C-reactive protein, gamma-glutamyl transferase, fever duration, and pleural effusion. An extended model based on eight features was evaluated as a sensitivity analysis. Final model selection was guided mainly by performance in temporal validation, while also taking into account discrimination stability and model simplicity. The resulting threshold was prespecified for model evaluation and should not be interpreted as a universal clinical decision cutoff.

### 2.8. Model Performance

In the development cohort (2015–2023), the five-feature random forest model showed good discriminatory performance, with an out-of-fold AUC of 0.895. When the prespecified threshold derived from the out-of-fold predictions (0.41) was applied, the sensitivity was 0.921 and the specificity was 0.828.

In the temporally independent 2024 cohort, the same model retained good discrimination, with an AUC of 0.854. At the same prespecified threshold, the sensitivity was 0.762 and the specificity was 0.812.

The other candidate algorithms showed broadly similar discrimination in the development cohort. In temporal validation, however, the random forest model achieved the highest AUC while maintaining a reasonable balance between sensitivity and specificity. The extended eight-feature model showed slightly higher discrimination during development, but this advantage was not sustained in the 2024 cohort. For this reason, and with consideration of model simplicity, the parsimonious five-feature random forest model was selected as the primary model [26].

### 2.9. Model Interpretability

Model interpretability was assessed for the final primary random forest model using SHAP. SHAP summary plots were generated to quantify how each predictor contributed to the model output. To provide a complementary measure of variable influence, feature importance was also assessed by permutation importance. These analyses were included to make the model more transparent and easier to interpret in a clinical context [27].

### 2.10. Ethics Approval

The study was approved by the Ethics Committee of the Children’s Hospital Affiliated to Chongqing Medical University (Approval No. 22, 2026).

## 3. Results

### 3.1. Study Population and Baseline Characteristics

Baseline characteristics of the development cohort (2015–2023) are presented in Table 1. The matched cohort included 227 children, comprising 76 necrotizing pneumonia cases and 151 non-necrotizing controls. Age, sex, and body weight were comparable between groups.

Several laboratory and clinical variables differed between groups. Children with necrotizing pneumonia had higher levels of CRP, D-dimer, ALT, and GGT. Fever duration was longer, and pleural effusion was more frequently observed among necrotizing cases. Prothrombin time and thrombin time showed overlapping distributions between groups. Baseline characteristics of the temporally independent 2024 validation cohort are provided in Supplementary Table S3.

### 3.2. Model Development and Discrimination

**Figure 2:**
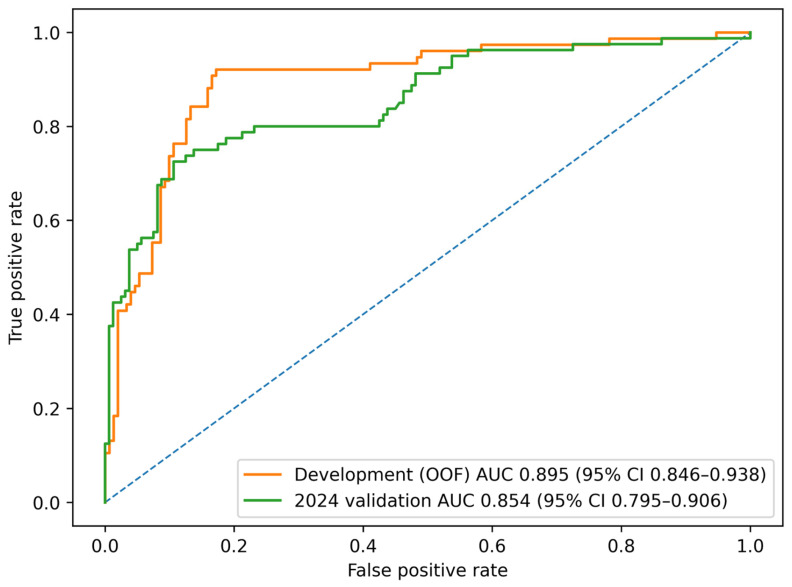
ROC curves for the final five-feature random forest model in the development cohort (2015–2023) and the temporally independent 2024 validation cohort. The out-of-fold AUC was 0.895 (95% CI, 0.846–0.938) in the development cohort, compared with 0.854 (95% CI, 0.795–0.906) in the 2024 validation cohort. The blue dashed line represents the reference line for a random classifier (AUC = 0.5).

Six machine-learning algorithms were compared in the development cohort. Among them, the random forest model showed the most stable overall performance when the development and temporal validation results were considered together, and it was therefore selected as the final primary model. Based on out-of-fold predictions from five-fold cross-validation, the five-feature random forest model achieved an AUC of 0.895 (95% CI, 0.846–0.938) in the development cohort. When the same model was applied to the temporally independent 2024 cohort, the AUC was 0.854 (95% CI, 0.795–0.906), indicating that its discriminatory ability was preserved in the later dataset (Figure 2).

At the prespecified threshold derived from the development cohort, the sensitivity and specificity in the 2024 validation cohort were 0.762 and 0.812, respectively. These findings support the temporal robustness of the model, although some decline in performance from the development cohort was observed, as expected. Detailed performance metrics for the final primary model are presented in Table 2.

The performance of the candidate machine-learning algorithms using the extended feature set is shown in Supplementary Table S4. Because the random forest model remained competitive in both datasets and provided the best performance in temporal validation, it was selected as the base model for subsequent comparison and interpretability analyses.

### 3.3. Calibration and Clinical Utility

**Figure 3:**
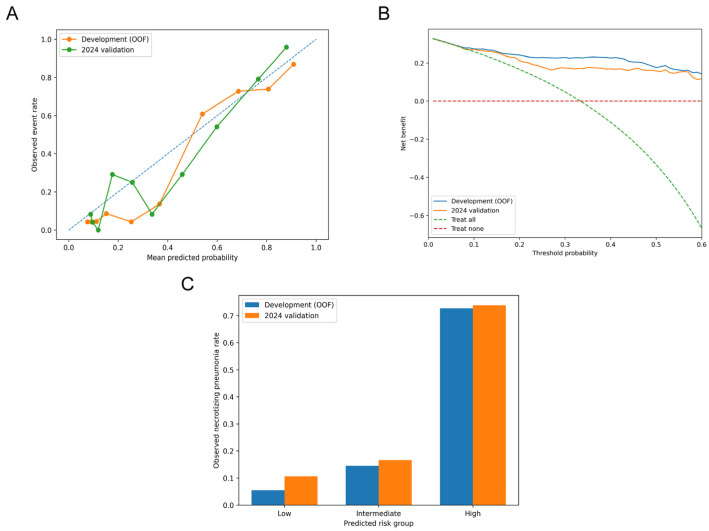
Calibration and decision-support performance of the primary prediction model. (**A**) Calibration plots for the development cohort and the 2024 temporal validation cohort, showing the relationship between predicted and observed risk across the range of predicted probabilities. The blue dashed diagonal line represents perfect calibration (predicted risk = observed risk). (**B**) Decision curve analysis comparing the net benefit of the model with the treat-all and treat-none strategies across threshold probabilities. (**C**) Observed event rates across the low-, intermediate-, and high-risk groups in the development and 2024 validation cohorts.

Calibration of the primary model is shown in Figure 3A. In the development cohort, the predicted probabilities were generally in reasonable agreement with the observed event rates across the range of risk. The Brier score was 0.121. The calibration intercept was −0.448 and the calibration slope was 1.258, which suggests a small degree of risk overestimation and some model optimism, although overall calibration remained acceptable [28].

In the 2024 validation cohort, calibration remained acceptable. The Brier score was 0.138. The calibration intercept was −0.239 and the calibration slope was 1.184. Although some deviation from ideal calibration was still present, the predicted risks generally tracked the observed event rates across the predefined risk strata.

Decision curve analysis is shown in Figure 3B. Across an approximate threshold range of 5% to 40%, the model yielded a higher net benefit than either the treat-all or treat-none strategy in both cohorts. The net benefit curves were broadly similar in the development and validation datasets, which suggests that the model retained potential decision-support value in the temporal validation cohort [29].

Risk stratification results are shown in Figure 3C. In the development cohort, the observed event rates were 5.5% in the low-risk group, 14.6% in the intermediate-risk group, and 72.7% in the high-risk group. In the 2024 cohort, the corresponding rates were 10.6%, 16.7%, and 73.8%, respectively. This gradient across the three risk groups was preserved in the temporally independent 2024 cohort, indicating that the model maintained a meaningful ability to separate lower-risk from higher-risk patients across time periods.

Taken together, these results suggest that the primary model combines good discrimination with acceptable calibration and retains potential decision-support value across the two time periods examined.

### 3.4. Model Interpretation

**Figure 4:**
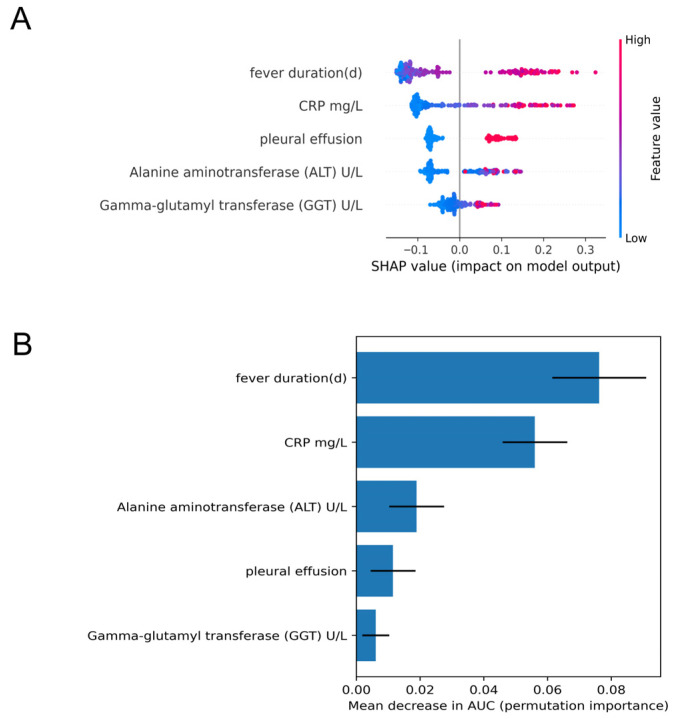
Model interpretation and predictor importance. (**A**) SHAP summary plot showing the direction and relative magnitude of each predictor’s contribution to the model output across individual patients. (**B**) Permutation importance analysis showing the contribution of each predictor to model discrimination, expressed as the decrease in AUC after random shuffling of the corresponding predictor values.

The SHAP summary plot is shown in Figure 4A and illustrates how each predictor contributed to the model output at the individual level. Among the five predictors, fever duration had the strongest influence on predicted risk. Longer fever duration was consistently associated with a higher predicted probability of necrotizing pneumonia. CRP also showed a clear positive association with model output. The presence of pleural effusion contributed substantially to increased predicted risk, whereas ALT and GGT showed smaller but directionally consistent effects.

Permutation importance analysis is presented in Figure 4B and provides a complementary view of predictor importance. Fever duration and CRP ranked highest, as random shuffling of either variable produced the largest decrease in AUC. Pleural effusion and ALT showed moderate importance, whereas GGT contributed less to model discrimination. The overall ranking was broadly consistent with the SHAP findings.

Taken together, these findings suggest that prolonged fever, systemic inflammatory activity, and pleural involvement were the main factors driving model predictions. The agreement between SHAP and permutation importance supports the internal consistency of the model and strengthens the clinical plausibility of the identified predictors.

### 3.5. Comparison Between Primary and Extended Models

We compared the predictive performance of the primary five-predictor model with that of the extended eight-predictor model using the same random forest framework (Supplementary Table S5).

In the development cohort, the extended model achieved a slightly higher AUC than the primary model. This advantage, however, was not maintained in the temporally independent 2024 validation cohort, where the extended model did not show improved discrimination. By comparison, the primary model showed more stable performance across the development and validation datasets. Because the two models performed similarly in temporal validation, the simpler five-predictor model was retained as the final model.

## 4. Discussion

In this propensity score–matched retrospective study, we developed and temporally validated a clinical prediction model for necrotizing pneumonia in children hospitalized with Mycoplasma pneumoniae pneumonia. Among the six candidate algorithms, the random forest model showed the most favorable overall balance between development and temporal validation performance and was therefore selected as the final primary model. In the development cohort, assessed by out-of-fold cross-validation, the model achieved an AUC of 0.895. In the temporally independent 2024 cohort, the AUC was 0.854. Taken together, these findings suggest that a small set of routinely available early variables may support risk stratification before overt necrotizing changes become apparent on imaging.

From a clinical perspective, the usefulness of a prediction model depends not only on discrimination, but also on whether the estimated risks are reasonably aligned with observed outcomes. In our study, calibration remained acceptable in temporal validation, which suggests that the predicted probabilities broadly tracked the observed event rates in the later cohort. Decision curve analysis provided additional support for potential clinical usefulness. Across an approximate threshold probability range of 5% to 40%, the model yielded greater net benefit than either the treat-all or treat-none strategy in both the development and validation cohorts. The broadly similar net benefit curves across the two periods suggest that this potential decision-support value was retained in temporal validation. Taken together, this pattern is more consistent with a model that may assist clinicians in identifying children who warrant closer monitoring, earlier reassessment, or more timely investigation once the predicted risk exceeds a clinically selected threshold.

The risk stratification results help translate these statistical properties into bedside language. When patients were grouped into low-, intermediate-, and high-risk strata, observed event rates increased stepwise and remained well separated in both datasets. In the development cohort, event rates were 5.5%, 14.6%, and 72.7% across the three strata; in the 2024 cohort, corresponding rates were 10.6%, 16.7%, and 73.8%. From a clinical perspective, this degree of separation suggests that the model may help identify children with meaningfully different levels of risk. Low-risk children may be less likely to need intensified evaluation based on concern for necrotizing progression alone, whereas high-risk children may represent a subgroup in whom earlier reassessment, closer monitoring, and more timely supportive investigation deserve greater consideration. The model is not intended to replace clinical judgment. Instead, it offers a structured way to direct attention toward children whose early symptoms and initial investigations have not yet evolved into the more typical radiologic picture of necrotizing pneumonia. Management decisions should still be individualized, with consideration of clinical trajectory, imaging findings, and local institutional protocols.

The interpretability analyses add another layer of support to the model findings by showing that the main predictors are clinically plausible. Across both SHAP values and permutation importance, fever duration emerged as the strongest contributor to predicted risk, followed by CRP, pleural effusion, ALT, and GGT. These predictors are also consistent with common clinical observations in severe or complicated MPP. Prolonged fever may reflect ongoing disease activity. Elevated CRP is in keeping with a stronger systemic inflammatory response, while pleural effusion may indicate more extensive pleuropulmonary involvement. ALT and GGT contributed less to the model output, but their effects were directionally consistent. This pattern may reflect broader inflammatory stress or extrapulmonary involvement in more severe infection. It is also important to note that SHAP was used here to improve model transparency and clinical interpretability, not to establish causal relationships. The associations identified by these analyses should therefore be interpreted as explanatory within the model rather than as evidence of causation.

We selected the five-predictor model rather than the eight-predictor model. Although the more complex model showed slightly better discrimination in the development dataset, this advantage was not maintained in the 2024 temporal validation cohort. In other words, the additional predictors did not improve external performance in the later dataset. This finding has practical implications. In routine clinical care, data may be incomplete, and decisions often need to be made under time pressure. Under these conditions, a smaller prediction model that retains acceptable performance in temporal validation may offer clear advantages. Our results support the view that, when external performance is comparable, a more parsimonious model may be more stable and easier to translate into clinical workflows. This may be particularly relevant when early risk assessment is needed in settings such as the emergency department or the inpatient ward.

These findings also help move routinely measured clinical indicators from a purely descriptive role into a predictive framework. Previous studies of pediatric necrotizing pneumonia have mainly focused on clinical characterization and retrospective analyses of associated risk factors. Reports from China consistently describe prolonged fever, elevated inflammatory markers, and frequent pleural effusion as common features in affected children [23,30,31]. In contrast, studies from other regions have often emphasized classic bacterial pathogens, such as *Streptococcus pneumoniae* and *Staphylococcus aureus*, as major causes of necrotizing pneumonia [15,16,32]. Evidence from China, however, increasingly suggests a shift in the epidemiology of pediatric necrotizing pneumonia, with Mycoplasma pneumoniae now recognized as a major cause, particularly in children aged three years and older [22,25,33]. Our findings are consistent with this trend. They also suggest that inflammatory and coagulation-related markers may have value within a predictive modeling framework, rather than serving only as descriptive indicators. Compared with traditional nomogram-based approaches or standard logistic regression models, which often rely on a limited number of predictors and may not always undergo extensive validation, the present model showed stable discrimination in both internal and temporally independent evaluations [25,33,34].

In practice, a model of this type may be most useful at the point when clinicians need to decide which children require closer attention. A higher predicted risk may support closer monitoring, earlier reassessment, or more timely imaging when the clinical picture is concerning. It may also help prompt multidisciplinary discussion in more complex cases. Conversely, a lower predicted risk may offer some reassurance when the clinical condition is improving, and it may help reduce unnecessary escalation driven mainly by uncertainty. Even so, any practical use of the model should remain embedded within established clinical pathways and safety safeguards, including repeated assessment based on clinical trajectory and physician concern. Several aspects of the study design lend support to the reliability of these findings. Propensity score matching was used to reduce baseline differences between groups, and multiple machine-learning algorithms were assessed in parallel rather than relying on a single modeling strategy. Model performance was examined from several perspectives, including discrimination, calibration, and decision curve analysis. Validation in a later cohort further allowed us to assess the temporal stability of the model.

Several limitations should be acknowledged. This was a single-center retrospective study, and its findings may not generalize fully to other practice settings or patient populations. Although temporal validation was performed using an independent 2024 cohort that was not involved in predictor screening or model development, this should still be regarded as temporal validation rather than true external validation, because all data came from the same institution. Independent evaluation in multicenter cohorts is therefore still required. Because necrotizing pneumonia was rare in the source population, model development relied on a propensity score–matched case–control framework. This approach improved comparability between cases and controls under rare-event conditions, but it does not fully reflect the real-world clinical setting in which physicians assess risk across the full spectrum of hospitalized children with MPP. For that reason, the predicted risks and thresholds reported here should be interpreted cautiously until they are tested, and if necessary recalibrated, in unselected cohorts. The model was also based on baseline or early in-hospital variables and did not account for dynamic changes in laboratory markers over time. A broader set of routine clinical and laboratory variables was examined during the initial analysis, but only predictors that met the predefined stability criteria across the four feature-selection methods were retained in the final model. Microbiological, resistance-related, genotypic, and more detailed radiologic features were not incorporated, and their added value should be explored in future studies. The classification threshold used in this study was selected statistically by maximizing the F1 score in the development cohort. It should therefore be interpreted as a prespecified evaluation cutoff rather than as a universal clinical decision threshold. Although calibration was acceptable in both cohorts, some deviation from ideal calibration remained, which further supports the need for external testing and possible recalibration in future work. We also did not evaluate the real-world impact of model-guided decision-making on management, outcomes, or resource use. Whether use of the model can reduce unnecessary escalation, improve outcomes, or support more efficient care pathways will need to be tested in prospective implementation studies.

Future work should focus on prospective multicenter validation in more diverse populations, so that model performance can be tested across different clinical settings and epidemiologic contexts. Incorporating microbiological data, host-response markers, and more detailed imaging features may further improve risk estimation. It will also be important to explore whether a streamlined version can be adapted for time-sensitive settings such as the emergency department. In the longer term, integration into electronic health record systems may support real-time risk alerts and repeated reassessment as new data become available. These next steps are needed before prediction modeling of this kind can be translated into routine clinical support for children at risk of necrotizing pneumonia.

## 5. Conclusions

We developed and temporally validated a machine learning–based prediction model for necrotizing pneumonia in children hospitalized with Mycoplasma pneumoniae pneumonia using routinely available early clinical data. The model showed good discrimination in both the development and later validation cohorts, which suggests that it may support early risk stratification before overt necrotizing changes become evident on imaging. This model is intended to complement, rather than replace, clinical judgment. Further external validation in independent multicenter cohorts is required before routine clinical implementation.

## Figures and Tables

**Table 1:** Baseline characteristics of the development cohort (2015–2023).

Variable	Non-Necrotizing Pneumonia (*N* = 151)	Necrotizing Pneumonia (*N* = 76)	*p* Value
Sex, *n* (%)			1.000
Male	68 (45.0%)	34 (44.7%)	
Female	83 (55.0%)	42 (55.3%)	
Age, months (mean ± SD)	77.4 ± 28.4	77.9 ± 29.9	0.906
Weight, kg, median (IQR)	21.0 (16.5, 26.0)	21.0 (16.8, 26.0)	0.997
ALT, U/L, median (IQR)	14.00 (11.00, 18.00)	28.00 (17.80, 46.25)	<0.001
CRP, mg/L, median (IQR)	14.6 (4.8, 31.0)	49.7 (21.5, 88.4)	<0.001
D-dimer, mg/L, median (IQR)	0.68 (0.33, 1.84)	3.10 (0.89, 6.72)	<0.001
GGT, U/L, median (IQR)	12.0 (9.0, 17.0)	15.5 (12.0, 32.3)	<0.001
Prothrombin time, s, median (IQR)	11.8 (11.3, 12.4)	11.9 (11.4, 12.6)	0.149
Thrombin time, s, median (IQR)	16.1 (15.6, 16.8)	16.2 (15.7, 16.9)	0.398
Fever duration, days, median (IQR)	7.0 (5.0, 8.5)	12.0 (9.0, 15.0)	<0.001
Pleural effusion, *n* (%)	25 (16.6%)	53 (69.7%)	<0.001

Notes: Values are presented as mean ± SD, median (IQR), or number (%).

**Table 2:** Performance of the final five-feature random forest model in the development and 2024 validation cohorts.

Cohort	AUC	Sensitivity	Specificity	PPV	NPV	F1
Development (OOF)	0.895	0.921	0.828	0.729	0.954	0.814
2024 validation	0.854	0.762	0.812	0.670	0.872	0.713

Notes: OOF refers to out-of-fold predictions generated during five-fold cross-validation in the 2015–2023 development cohort. The decision threshold was selected in the development cohort by maximizing the F1 score and was then applied without modification to the 2024 validation cohort.

## Data Availability

The datasets generated and analyzed during this study are not publicly available due to ethical and data protection restrictions. De-identified data may be available from the corresponding author upon reasonable request, subject to institutional ethics approval and completion of a data sharing agreement. The code supporting the findings is openly available at https://github.com/lilylu-hub/paper-code (accessed on 25 January 2026).

## Supplementary Materials

The following supporting information can be downloaded at: https://www.mdpi.com/article/10.3390/children13040473/s1, Table S1: Missing data in development and validation cohorts; Table S2: Initial candidate predictors considered for feature selection; Table S3: Baseline characteristics of the temporal validation cohort; Table S4: Performance of the extended eight-feature model in development (OOF) and temporal validation; Table S5: Comparison of primary and extended feature sets using the random forest model.

## Abbreviations

The following abbreviations are used in this manuscript:

MP-IgM	Mycoplasma pneumoniae immunoglobulin M
MP-IgG	Mycoplasma pneumoniae immunoglobulin G
F1	F1 score
ALT	Alanine aminotransferase
AUC	Area under the curve
CI	Confidence interval
CRP	C-reactive protein
GGT	Gamma-glutamyl transferase
MPP	*Mycoplasma pneumoniae* pneumonia
NP	Necrotizing pneumonia
MPNP	MPP-associated necrotizing pneumonia
OOF	Out-of-fold
PCR	Polymerase chain reaction
PT	Prothrombin time
ROC	Receiver operating characteristic
SHAP	SHapley Additive exPlanations
TT	Thrombin time
