# Supplementary material for: Early Prediction of Necrotizing Pneumonia in Children with Mycoplasma Pneumoniae Pneumonia: Development and Temporal Validation of a Clinical Model
Source: Children (Basel). 2026 Mar 29;13(4):473. doi: 10.3390/children13040473 (PMC13115073; doi:10.3390/children13040473)
Supplement: Supplementary file 1 [file children-13-00473-s001.zip › Supplementary Table S1. Missing data in development and validation cohorts.pdf]

Supplementary Table S1. Missing data in development and validation cohorts

Variable	Development (n=227), n (%)	Validation 2024 (n=240), n (%)
Pleural effusion	0 (0.0%)	0 (0.0%)
CRP (mg/L)	1 (0.4%)	3 (1.3%)
ALT (U/L)	2 (0.9%)	1 (0.4%)
GGT (U/L)	2 (0.9%)	1 (0.4%)
Fever duration (days)	1 (0.4%)	1 (0.4%)
D-dimer (mg/L)	2 (0.9%)	0 (0.0%)
PT (s)	0 (0.0%)	0 (0.0%)
TT (s)	0 (0.0%)	0 (0.0%)
