# Supplementary material for: Early Prediction of Necrotizing Pneumonia in Children with Mycoplasma Pneumoniae Pneumonia: Development and Temporal Validation of a Clinical Model
Source: Children (Basel). 2026 Mar 29;13(4):473. doi: 10.3390/children13040473 (PMC13115073; doi:10.3390/children13040473)
Supplement: Supplementary file 1 [file children-13-00473-s001.zip › Supplementary Table S2. Initial candidate predictors considered for feature selection.pdf]

Supplementary Table S2. Initial candidate predictors considered for feature selection

Variable	Category	Form in analysis	Retained in extended model	Retained in primary model
Peak fever temperature (℃)	Clinical	Continuous	No	No
Fever duration (d)	Clinical	Continuous	Yes	Yes
Coexisting bacterial infection	Clinical	Binary	No	No
Atelectasis	Imaging	Binary	No	No
Pleural effusion	Imaging	Binary	Yes	Yes
PCT (ng/mL)	Laboratory	Continuous	No	No
CRP (mg/L)	Laboratory	Continuous	Yes	Yes
WBC (×10^9/L)	Laboratory	Continuous	No	No
RBC (×10^12/L)	Laboratory	Continuous	No	No
HB (g/L)	Laboratory	Continuous	No	No
NEUT (%)	Laboratory	Continuous	No	No
LYMPH (%)	Laboratory	Continuous	No	No
D-dimer (mg/L)	Laboratory	Continuous	Yes	No
Fibrinogen (g/L)	Laboratory	Continuous	No	No
Prothrombin time (PT) (s)	Laboratory	Continuous	Yes	No
Thrombin time (TT) (s)	Laboratory	Continuous	Yes	No
Direct bilirubin (DBIL) (μmol/L)	Laboratory	Continuous	No	No
Albumin (ALB) (g/L)	Laboratory	Continuous	No	No

Variable	Category	Form in analysis	Retained in extended model	Retained in primary model
Alanine aminotransferase (ALT) (U/L)	Laboratory	Continuous	Yes	Yes
Gamma-glutamyl transferase (GGT) (U/L)	Laboratory	Continuous	Yes	Yes
Lactate dehydrogenase (LDH) (U/L)	Laboratory	Continuous	No	No
Cholinesterase (CHE) (U/L)	Laboratory	Continuous	No	No
Uric acid (UA) (μmol/L)	Laboratory	Continuous	No	No
Chloride (Cl^-) (mmol/L)	Laboratory	Continuous	No	No
Calcium (Ca^{2+})	Laboratory	Continuous	No	No

All variables listed in Supplementary Table S1 were available during the early hospitalization period and were entered into the initial candidate set for feature selection in the development cohort (2015–2023). Variables retained by at least three of the four feature-selection methods were included in the extended model. Variables retained by all four methods were included in the final primary model.
