# Supplementary material for: Early Prediction of Necrotizing Pneumonia in Children with Mycoplasma Pneumoniae Pneumonia: Development and Temporal Validation of a Clinical Model
Source: Children (Basel). 2026 Mar 29;13(4):473. doi: 10.3390/children13040473 (PMC13115073; doi:10.3390/children13040473)
Supplement: Supplementary file 1 [file children-13-00473-s001.zip › Supplementary Table S3. Baseline characteristics of the temporal validation cohort.pdf]

Table S3. Baseline characteristics of the temporal validation cohort

Variable	Non-necrotizing pneumonia (N = 160)	Necrotizing pneumonia (N = 80)	P value
Sex, n (%)			1.000
Male	72 (45.0%)	36 (45%)	
Female	88 (55.0%)	44 (55%)	
Age, months, median (IQR)	80.2 (59.0, 92.4)	83.5 (61.0, 92.5)	0.674
Weight, kg, median (IQR)	20.00 (17.00, 25.00)	20.75 (17.00, 25.50)	0.496
ALT, U/L, median (IQR)	14.00 (11.00, 18.00)	28.00 (17.80, 46.25)	<0.001
CRP, mg/L, median (IQR)	10.91 (4.89, 25.54)	41.20 (20.40, 89.09)	<0.001
D-dimer, mg/L, median (IQR)	0.47 (0.32, 0.77)	2.72 (0.80, 7.58)	<0.001
GGT, U/L, median (IQR)	11.00 (9.00, 13.00)	13.00 (11.00, 24.25)	<0.001
Prothrombin time, s, median (IQR)	11.30 (10.90, 11.90)	11.95 (11.30, 12.53)	<0.001
Thrombin time, s, median (IQR)	16.80 (16.20, 17.40)	16.20 (15.70, 16.90)	<0.001
Fever duration, days, median (IQR)	6.00 (4.00, 8.00)	10.00 (7.00, 12.25)	<0.001
Pleural effusion, n (%)	23 (14.4%)	40 (50%)	<0.001

Notes: Values are presented as mean ± SD, median (IQR), or number (%).

CRP, C-reactive protein; ALT, Alanine aminotransferase; GGT, gamma-glutamyl transferase;
