# Supplementary material for: Early Prediction of Necrotizing Pneumonia in Children with Mycoplasma Pneumoniae Pneumonia: Development and Temporal Validation of a Clinical Model
Source: Children (Basel). 2026 Mar 29;13(4):473. doi: 10.3390/children13040473 (PMC13115073; doi:10.3390/children13040473)
Supplement: Supplementary file 1 [file children-13-00473-s001.zip › Supplementary Table S4. Performance of the extended eight-feature model in development (OOF) and temporal validation.pdf]

Table S4. Performance of candidate algorithms using the extended eight-feature set in development (OOF) and temporal validation (2024)

Model	Threshold (OOF)	AUC (OOF)	Sens (OOF)	Spec (OOF)	F1 (OOF)	AUC (2024)	Sens (2024)	Spec (2024)	F1 (2024)
Logistic regression	0.41	0.930	0.868	0.868	0.815	0.791	0.650	0.788	0.627
Random forest	0.41	0.916	0.868	0.874	0.820	0.831	0.688	0.881	0.714
XGBoost	0.28	0.909	0.882	0.848	0.807	0.824	0.725	0.819	0.695
SVM	0.32	0.926	0.842	0.848	0.785	0.799	0.625	0.844	0.645
KNN	0.21	0.894	0.789	0.854	0.759	0.804	0.650	0.863	0.675
Decision tree	0.43	0.877	0.882	0.815	0.784	0.783	0.725	0.756	0.655

Notes: Thresholds were derived in the development cohort using out-of-fold (OOF) predictions by maximizing the F1 score and were applied unchanged to the 2024 cohort. OOF indicates five-fold cross-validation out-of-fold performance.

Extended model predictors (8): ALT; CRP; D-dimer; GGT; PT; TT; fever duration; pleural effusion.

Abbreviations: AUC, area under the receiver operating characteristic curve; KNN, k-nearest neighbors; OOF, out-of-fold; Sens, sensitivity; Spec, specificity; SVM, support vector machine.
