# Supplementary material for: Early Prediction of Necrotizing Pneumonia in Children with Mycoplasma Pneumoniae Pneumonia: Development and Temporal Validation of a Clinical Model
Source: Children (Basel). 2026 Mar 29;13(4):473. doi: 10.3390/children13040473 (PMC13115073; doi:10.3390/children13040473)
Supplement: Supplementary file 1 [file children-13-00473-s001.zip › Supplementary Table S5. Comparison of primary and extended feature sets using the random forest model.pdf]

Table S5. Comparison of primary and extended feature sets using the random forest model

Feature set	No. of predictors	AUC (Development, OOF)	AUC (2024 validation)
Primary (5 features)	5	0.895	0.854
Extended (8 features)	8	0.916	0.831

Notes: AUC values in the development cohort were based on out-of-fold predictions from five-fold cross-validation. Temporal validation was performed using the independent 2024 cohort.

Primary predictors (5): ALT; CRP; GGT; fever duration; pleural effusion.

Extended predictors (8): ALT; CRP; D-dimer; GGT; PT; TT; fever duration; pleural effusion.

Abbreviations: AUC, area under the receiver operating characteristic curve; OOF, out-of-fold; ALT, alanine aminotransferase; CRP, C-reactive protein; GGT, gamma-glutamyl transferase; PT, prothrombin time; TT, thrombin time.
